# Setting priorities for development of emerging interventions against childhood diarrhoea

**DOI:** 10.7189/jogh.03.010302

**Published:** 2013-06

**Authors:** Zulfiqar A. Bhutta, Alvin Zipursky, Kerri Wazny, Myron M. Levine, Robert E. Black, Diego G. Bassani, Mathuram Shantosham, Stephen B. Freedman, Adenike Grange, Margaret Kosek, William Keenan, William Petri, Harry Campbell, Igor Rudan

**Affiliations:** 1 Program for Global Pediatric Research, the Hospital for Sick Children, Toronto, Canada; 2 Division of Women and Child Health, Aga Khan University Hospital, Karachi, Pakistan; 3 Center for Vaccine Development, University of Maryland School of Medicine, Baltimore, MD, USA; 4 Department of International Health, Bloomberg School of Public Health, Johns Hopkins University, Baltimore, MD, USA; 5 Department of Pediatrics, Faculty of Medicine, University of Toronto, Toronto, Ontario, Canada; 6 Alberta Children’s Hospital Research Institute, University of Calgary, Calgary, Alberta, Canada; 7 International Pediatrics Association, Elk Grove Village, IL, USA; 8 St. Louis University, St. Louis, MO, USA; 9 University of Virginia, Charlottesville, VA, USA; 10 Centre for Population Health Sciences and Global Health Academy, The University of Edinburgh Medical School, Scotland, UK

Diarrhoeal diseases are still among the leading causes of childhood mortality in the world, contributing to more than 800 000 deaths in children younger than 5 years of age in 2010 [1,2]. It is widely acknowledged that a major portion of those deaths can be prevented if universal coverage of known effective interventions could be achieved [3,4]. However, recent evaluations have shown that the uptake of those interventions is rather disappointing, with only a minority of all children with life–threatening episodes of diarrhoea in low– and middle–income countries having access to trained health care providers and receiving appropriate treatment [5–7]. Thus, novel diarrhoea control strategies that balance investments in scaling up of existing interventions and the development of novel approaches, technologies and ideas are needed.

The importance of childhood diarrhoea as a global public health problem is in stark contrast to the size of the research community focused on the issue and the amount of funding committed to studying the disease [8]. However, there have been several efforts in recent years that aimed to mobilize the research community and encourage research on childhood diarrhoea. Kosek et al. used the Child Health and Nutrition Research Initiative (CHNRI) methodology to define research priorities that could immediately reduce the burden of disease [9]. This was followed by the World Health Organization's CHNRI exercise that defined research priorities to reduce global mortality from childhood diarrhoea by the year 2015, ie, the time of the fourth Millennium Development Goal's target [10]. Recently, a new initiative has been launched – Global Action Plan for Pneumonia and Diarrhoea (GAPPD) – which prioritizes a research agenda for childhood diarrhoea beyond 2015 [2–4,11]. The results of their very comprehensive CHNRI process, with the timeline extended 15 years ahead and an expanded scope of research on preventing morbidity, have been published recently[12]. The purpose of this exercise was to further contribute to the active field of setting research priorities for childhood diarrhoea, by reviewing the landscape of new ideas and/or novel potential interventions – hereby referred to as “emerging interventions” – and set priorities for investments in their development.

## EMERGING INTERVENTIONS AGAINST CHILDHOOD DIARRHOEA

The 10 emerging interventions were chosen for evaluation from the results of a previous CHNRI exercise [12] and further consultation with a paediatric gastroenterologist (**Table 1**).The previous exercise [12] brought together 10 teams, corresponding to research avenues in childhood diarrhoeal disease. Each team separately generated and ranked their set of research questions. While one team focused specifically on emerging interventions, many teams generated research priorities related to emerging interventions and thus, we considered novel interventions proposed by all teams for inclusion in this exercise. We aimed to be open–minded and inclusive in the selection of these interventions, appreciating that some of them may still need considerable work before being ready for implementation.

**Table 1:** The consolidated list of 10 emerging interventions against childhood diarrhoea

1. Probiotics and prebiotics
2. Anti–emetics
3. Treatment or prevention (vaccines) of environmental enteropathy
4. Sustainable, affordable latrine options
5. Household– or community–level water treatment
6. CFTR inhibitors
7. Inhibitors of intestinal epithelial function in the treatment of diarrhoea
8. Antibiotic therapy of *Cryptosporidium* diarrhoea
9. Oral or transcutaneous vaccine development
10. Combination vaccine for *Cryptosporidium*, *Shigella*, and enterotoxigenic *Escherichia coli* (ETEC)

CFTR – cystic fibrosis transmembrane conductance regulator

## EXPERT OPINION EXERCISE

The CHNRI methodology for priority setting in investments in health research and technologies was proposed as a systematic tool that can be used to develop research policy and/or prioritize investments in health research [13–14]. The CHNRI method consists of the following steps: (i) investors and policy–makers define the context of the problem and identify the criteria for priority–setting; (ii) technical experts generate research priorities and score them against the pre–defined criteria; and, (iii) other stakeholders decide on the weight of the criteria, intended to reflect a wider societal system of values. The method has been described elsewhere, and many examples of implementation have been published [15–18].

A group of 12 leading international experts were invited to participate in the expert opinion exercise. The group was instructed to use a downstream (ie, broad, long–term) approach and focus solely on emerging interventions. During October 2012 the group ranked 10 emerging interventions according to a number of criteria used to identify priorities for research support in the area of childhood diarrhoea. A modified version of CHNRI’s conceptual framework (**Table 2**) was used and included 12 criteria for prioritization of emerging interventions: 1) answerability (in an ethical way); 2) low development cost; 3) low product cost; 4) low implementation cost; 5) predicted efficacy; 6) likelihood of deliverability; 7) likelihood of affordability; 8) likelihood of sustainability; 9) maximum potential impact on mortality burden reduction; 10) likelihood of acceptability to health workers; 11) likelihood of acceptability to end users; 12) predicted impact on equity [19].

**Table 2:** Specific questions used to assess whether the proposed research themes (eg, emerging interventions) satisfy the 12 priority–setting criteria

**1. Answerability in an ethical way** *(“1” for Yes;“0” for No;“0.5” for Undecided)*
▪ Do we have a sufficient research and development capacity to make the intervention available on the market by 2025?
▪ Do we have a sufficient level of funding support to make the intervention available on the market by 2025?
▪ Would you say that it is likely that the remaining technical hurdles can be overcome to make the intervention available on the market by 2025?
**2. Low development cost**(*“1” for Yes;“0” for N;“0.5” for Undecided)*
▪ How much will it cost to get from the current stage of development to commercial availability of each emerging intervention below?
a. <US$ 1 billion b. <US$ 500 million c. <US$ 100 million
**3. Low product cost**(*“1” for Yes;“0” for N;“0.5” for Undecided)*
▪ Is it likely to be a low–cost intervention (ie, <US$ 3.50 per unit?)
**4. Low implementation cost**(*“1” for Yes; “0” for N; “0.5” for Undecided)*
▪ Can we use the existing delivery mechanisms without major modifications (eg, training, infrastructure)?
**5. Affordability**(*“1” for Yes; “0” for N; “0.5” for Undecided)*
▪ Is achievement of a near–universal coverage likely to be affordable to most developing countries?
**6. Predicted efficacy** (*0–100%)*
▪ Please assess the likelihood (0–100%) that adequately powered randomized controlled trials of the interventions conducted in developing countries would consistently show statistically significant reduction in cause–specific mortality from childhood diarrhoea.
**7. Likely maximum potential impact on mortality burden**
▪ Please predict the proportion of deaths in children under 5 years of age due to diarrhoea that could be averted if the complete coverage with the emerging interventions listed below could be achieved?
**8–9. Deliverability and Sustainability** (*“1” for Yes;“0” for N;“0.5” for Undecided)*
▪ Taking into account (i) the infrastructure and resources required to deliver emerging interventions listed below (eg, human resources, health facilities, communication and transport infrastructure); (ii) the resources likely to be available to implement the emerging interventions at the time of introduction; (iii) overall capacity of the governments (eg, adequacy of government regulation, monitoring and enforcement; governmental intersectoral coordination), and (iv) internal and external partnership required for delivery of interventions (eg, partnership with civil society and external donor agencies), would you say that the emerging interventions would be: a. Deliverable at the time of introduction? b. Sustainable for at least 10 years after the time of introduction?
**10–12. Acceptability to health workers; Acceptability to end–users; and Impact on equity** (*“1” for Yes;“0” for N;“0.5” for Undecided)*
▪ Taking into account the overall context, intervention complexity, health workers’ behaviour and the end–user population at the time of introduction, a. Would health workers be likely to comply with implementation guidelines? b. Would end–users be likely to fully accept the intervention? c. Would you say that the proposed intervention has the overall potential to improve equity after 10 years following the introduction?

All experts invited to participate in the exercise received a brief (1 page) background document containing information on each of the 10 emerging interventions. This document also explained why each of the 12 criteria was chosen and how to apply them to each emerging intervention. The experts were free to challenge all information provided to them in a background document and to share further personal knowledge or opinion with the group. The experts were invited by e–mail to score, independently of each other, all emerging interventions according to the 12 agreed CHNRI criteria.

The scoring of the emerging interventions was conducted using a points system that assigned a value of 1 to positive answers (yes) and 0 to negative answers (no). When an expert assigned the answer “undecided” to a given criteria, the value of 0.5 points was used. Conversely, when the expert declared to be insufficiently informed on a given issue to answer the question, the input was deemed missing and not scored (or penalized). Each research question/theme received a score for all 12 criteria from each expert and the final score was calculated as the average of the ratios of the sum of all points given over the maximum possible number of points (excluding missing inputs). Such final score ranged from 0 to 100% and represents a direct measure of ‘collective optimism’ for a given emerging intervention.

**Figure:**
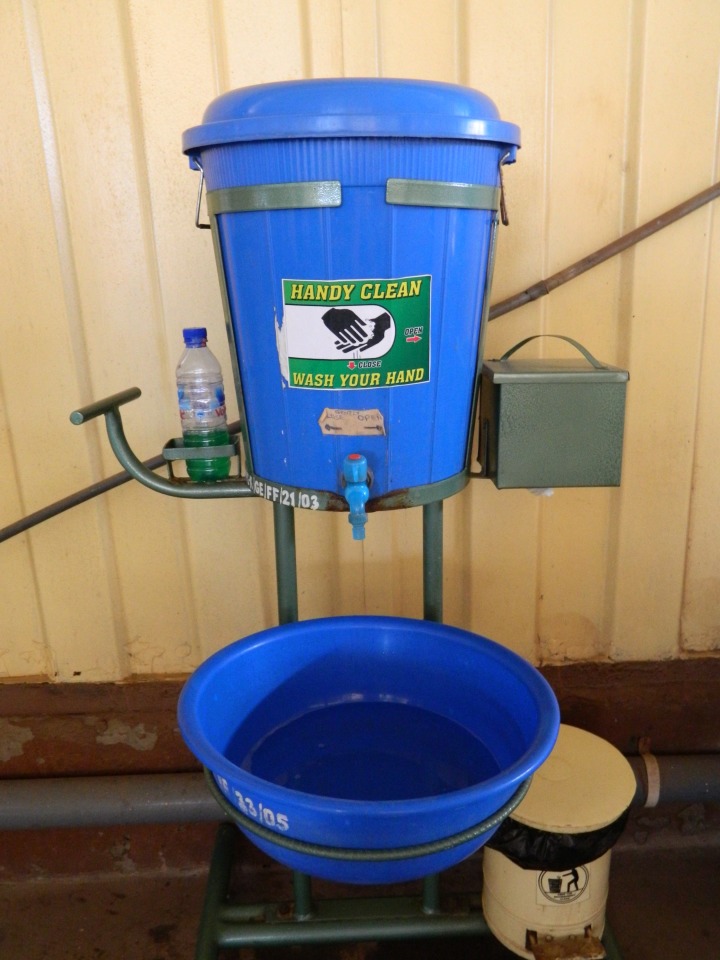
Photo: Courtesy of Alice Graham, personal collection

In the initial exercise, we included different components of the cost (development cost, product cost, implementation cost and affordability), but those 4 criteria are in fact a single criterion (cost). We therefore decided to exclude 3 criteria to ensure the remaining criteria were relatively independent of one another (similar to principal component analysis). If all 4 were kept in the exercise, this would give an undue four–fold “weight” to one criterion at the expense of the others. The experts agreed that the most important of the 4 cost–related criteria related to emerging interventions is “development cost”, because costs of product and implementation can be met through other mechanisms (such as GAVI, PEPFAR, Global Fund, etc.). Thus, the cost of product, cost of implementation and affordability were kept out of the final score calculation. After the exclusion, mean scores given to each criterion by the experts was calculated. The overall research priority score (RPS) was calculated as a mean of the 9 intermediate criteria scores. The scores given to all 10 emerging interventions are presented in **Table 3**.

**Table 3:** The results of the CHNRI exercise: 10 emerging interventions with 9 intermediate scores and an overall research priority score (RPS)

Rank	Emerging intervention	Answerability	Low development cost	Likelihood of efficacy	Max burden reduction potential	Deliverable	Sustainable	Acceptable to health workers	Acceptable to end users	Impact on equity	Research investment priority score
1	Household– or community–level water treatment	78	47	100	44	68	77	95	86	100	77.3
2	Sustainable, affordable latrine options	64	60	95	45	41	86	91	95	91	74.4
3	Antibiotic therapy of *Cryptosporidium* diarrhoea	72	49	82	23	64	77	86	91	70	68.2
4	Oral or transcutaneous vaccine development	44	36	77	39	64	64	100	95	82	66.9
5	Probiotics and prebiotics	74	54	60	10	59	86	77	86	64	63.5
6	*Cryptosporidium*, *Shigella*, and enterotoxigenic *Escherichia coli* (ETEC)	42	29	82	33	55	68	100	86	73	63.1
7	Anti–emetics	65	50	59	7	64	67	91	95	45	60.4
8	Treatment or prevention (vaccines) of environmental enteropathy	29	53	41	39	45	41	80	60	60	49.9
9	CFTR inhibitors	35	42	55	9	15	30	61	61	28	37.4
10	Inhibitors of intestinal epithelial function in the treatment of diarrhoea	38	36	45	21	36	36	44	56	39	39.1

CFTR – cystic fibrosis transmembrane conductance regulator

## MAIN FINDINGS

As shown in **Table 3**, the panel declared most of their collective optimism towards developing household– or community–level water treatment, followed by sustainable, affordable latrine options. The key strengths of those interventions were high likelihood of efficacy, acceptability both among health workers and end–users, and positive impact on equity in the population. Those two interventions were followed by antibiotic therapy for *Cryptosporidium* diarrhoea, and oral or transcutaneous enteric vaccine development. These two emerging interventions had similar scores as the two top–ranked, but their impact on equity in the population was deemed less certain.

The second priority level was assigned to probiotics and prebiotics; combination vaccine for *Cryptosporidium*, *Shigella*, and enterotoxigenic *Escherichia coli* (ETEC); and the use of anti–emetics in diarrhoea case management. The key weaknesses of the combination vaccine were uncertain answerability and high predicted cost of development, while the key uncertainty over the use of probiotics and anti–emetics is related to very low optimism towards their potential for the reduction of the overall burden of diarrhoea.

The lowest level of optimism was expressed towards the treatment or prevention of environmental enteropathy, cystic fibrosis transmembrane conductance regulator (CFTR) inhibitors, and the use of inhibitors of intestinal epithelial function in the treatment of diarrhoea. These three interventions had uncertain answerability and effectiveness, with low scores on deliverability, sustainability and acceptability. These interventions were also deemed to have negative impact on equity following their implementation (**Table 3**).

## CONCLUSION

In contrast to very large significance that childhood diarrhoea still has as a public health problem in low and middle income countries, this exercise suggested that relatively few novel interventions can be considered feasible at this point. The experts were most optimistic about the potential for Water and Sanitation Hygiene (WASH) interventions to be efficacious and reduce inequities. Although there are high development costs associated with most of the emerging interventions, investments in this sector will help reduce inequities and reduce the burden of childhood diarrhoea. In synergy with powerful interventions that are currently available, diarrhoeal mortality could be significantly reduced in the next 15 years.
